# Supplementary material for: The effects of fasting on acute ischemic infarcts in the rat
Source: PLoS One. 2024 Dec 12;19(12):e0307313. doi: 10.1371/journal.pone.0307313 (PMC11637405; doi:10.1371/journal.pone.0307313)
Supplement: Attachment — Submitted filename: Response to Reviewers.docx [file pone.0307313.s002.docx]

**Response to reviewers**

Manuscript ID: PONE-D-23-03308

Manuscript title: The effects of fasting on ischemic infarcts in the rat

We want to thank the editor for the thoughtful feedback and hope we could improve our manuscript by addressing the insightful comments. Please find attached our point-by-point response to each of the comments in the previous decision letter.

**Comment 1** The photos are in black and white, making it impossible to compare them with the controls. They should be replaced with color photos.

**Response**: All images were changed from black-and-white to color.

**Comment 2**: There are insufficient descriptions in the methods. There is no description of the method and amount of blood collected for the determination of blood glucose and ketones. Ketone bodies are not described as acetone, beta-hydroxybutyrate, or acetoacetic acid.

**Response**: We added more detail to the methodology section on blood glucose and ketone body measurement. The beginning of the corresponding paragraph now reads as follows: “After 24 hours from the start of treatment, rats were deeply anaesthetized with 5% isoflurane in 70% N_2_ and 30% O_2_ and their weight was assessed. Their blood glucose and β-hydroxybutyrate ketone levels were measured. This was done by using blood from a finger prick (minimum 1.2 μl) that was immediately applied onto a set strip and inserted into the glucose- and ketone body meter through which the results could be read off within 10 seconds (On Call GK Dual Blood Glucose & Ketone Monitoring System, Acon Laboratories, San Diego, CA, USA).”
