# Supplementary material for: The effects of fasting on acute ischemic infarcts in the rat
Source: PLoS One. 2024 Dec 12;19(12):e0307313. doi: 10.1371/journal.pone.0307313 (PMC11637405; doi:10.1371/journal.pone.0307313)
Supplement: Attachment — Submitted filename: 18.11.2023 Response to Reviewers Comments.docx [file pone.0307313.s003.docx]

**Response to Reviewers Comments**

Reviewer #1:

1. Estimating infarct volume at 1 day is not sufficient in ischemia studies. They need to conduct longer term studies to confirm that effects of fasting are long-lasting. This important as mice eat less food during the first day after ischemia.

We thank the reviewer for taking the time to carefully review our manuscript. Whilst we agree that it is important, especially in the context of the long-term consequences of stroke, to determine the effects of any interventions on chronic outcomes of ischemia we would also like to highlight that was not the main aim of this study. This preliminary investigation aimed only to determine the effects of fasting on the acute phases post-ischemia to determine whether a longer study would prove beneficial. We would also be keen for the reviewer to note that the model used is the endothelin model in rats, not mice. This model has been used because it produces a particularly robust, reproducible and – most importantly – mild injury. The aim was to study a defined infarct area in the first instance. This model produces limited behavioral deficits and no obvious hypophagia in the first 24 hours (1, 2). Ongoing studies in our laboratory aim to look at the chronic effects of acute fasting and caloric restriction on the outcomes of ischemia but these were not the scope of the current study. We feel that it remains important for scientific collaboration, reproducibility and progress that data are published when they form a complete story. This study aimed to investigate the acute effects of fasting on the acute stages of injury. In line with this, we have adjusted the title to include the word ‘acute’ to make this clear.

2. The scope of this study is very skimpy. Outcomes are disappointingly low in number. They need to show if fasting leads to neurological benefits by studying at least motor function after ischemia.

As mentioned above, this study aimed to investigate the acute effects of fasting on the molecular biology of the infarct. We have highlighted this in our adjusted title. The ongoing work in our laboratory aims to investigate the role of fasting on more detailed behavioural outcomes such as paw reaching, motor learning and neurological scoring. We also aim to use other models of stroke, including the middle cerebral artery occlusion model, which produces more significant behavioral deficits than the endothelin model and as such would be more amenable to this kind of testing. As we hope the reviewer appreciates, many projects are produced with limited time and funds and must be published to secure further funding to be expanded. This is a preliminary data set that we feel would benefit the field of stroke research.

3. The rigor of the data is low. In Fig. 1, infarct volume in control group was spread from 1 % to 12% in various animals. But, the SEM was shown as only ~1%.

As noted by previous studies from our group as well as others, within-group variability of stroke volume has been discussed extensively in the literature. The reasons for this include the collateral system of the animal studied and the energy availability at the time point of study, amongst others. Since the SEM quantifies uncertainty in estimates of the mean, the SD indicates the actual dispersion of the data from the mean. Accordingly, one can still have a relatively high variability in the data with a low SEM. We have, based on comment number 7 and in accordance with the current statistical reporting guidelines, amended the reporting from using SEM to SD.

4. Rigor of immunostaining is poor. The Iba-1 stained and GFAP stained images are not acceptable for counting the cell number. I can’t see any cells in the sections they provided.
5. For Fig. 1, it is not sufficient to give one CV-stained section for each group. Provide serial brain sections for at least one animal of each group.

We appreciate the reviewer’s comments. Whilst many groups do use serially stained sections using TTC, many use cresyl violet, toluidine blue or haematoxylin and provide a single image as an example of maximum infarct volume, this is standard within the field of pre-clinical stroke research. Some examples are provided in the table below. It should be noted that within this list, researchers such as Kate Lambertsen are prominent within the field of stroke research and regularly represent their lesions in this way.

| Sudhir Karthikeyan et al 2018 | Characterizing Spontaneous Motor Recovery Following Cortical and Subcortical Stroke in the Rat. |
| --- | --- |
| Bettina Hjelm Clausen et al 2017 | Fumarate decreases edema volume and improves functional outcome after experimental stroke. |
| Bettina Hjelm Clausen et al 2016 | Conditional ablation of myeloid TNF increases lesion volume after experimental stroke in mice, possibly via altered ERK1/2 signaling. |
| Hima C. S. Abeysinghe et al 2014 | Brain Remodelling following Endothelin-1 Induced Stroke in Conscious Rats. |
| Hiroshi Kamada et al 2007 | Bad as a Converging Signaling Molecule between Survival PI3-K/Akt and Death JNK in Neurons after Transient Focal Cerebral Ischemia in Rats. |
| Kate Lykke Lambertsen et al 2004 | A Role for Interferon-Gamma in Focal Cerebral Ischemia in Mice. |
| Kate Lykke Lambertsen et al 2004 | A Quantitative Study of Microglial—Macrophage Synthesis of Tumor Necrosis Factor during Acute and Late Focal Cerebral Ischemia in Mice. |
| Kate Lykke Lambertsen et al 2002 | Microglial—Macrophage Synthesis of Tumor Necrosis Factor after Focal Cerebral Ischemia in Mice is Strain Dependent. |

6. Need to present infarct volume as mm3 rather than % of ipsilateral hemisphere.

In our previous studies, we have used MRI as a means to analyse infarct volume. As part of these studies, and in studies by others it has been noted that rat brain volume varies by body weight and as such, calculating lesion volume as a percentage of the rats' own brain proved to be an effective way to counter this variability and controls for inter-individual differences in brain volumes (3). This also allows analysis to take into account edema and was originally published by Swanson in 1990 (4).

7. It is a better practice to provide SD than SEM.

We thank the reviewer for this comment and adjusted the reporting of the statistical analysis from SEM to SD accordingly. Please also see our response to comment 3 for further justification.

Reviewer #2:

This study explores the intriguing and significant impact of a 24-hour fasting regimen on inflammation and brain injury induced by stroke. The research has been conducted with meticulous attention; however, this reviewer would like to offer a few suggestions for further enhancing the study prior to publication. Critique:

1. Incorporating dietary interventions before the occurrence of brain injury holds translational relevance, as it has been demonstrated to prime the organism for protection against major stress conditions. While post-treatment (fasting) serves as an intervention, pre-treatment acts as a preventive measure. Both approaches hold translational significance.

We thank the reviewer for their stimulating comment. We agree that there is some degree of translational relevance in that fasting as a matter of habit may improve recovery if the individual were to have a stroke, our main goal is to help those who have already had a stroke, irrespective of their prior dietary habits. We have highlighted this in the text and adjusted to make this clear, and pointed out that pre-stroke fasting may be a beneficial preventative measure: “Most studies introduced the dietary intervention before the brain injury, which highlights its potential as a preventative measure. This study, however, introduces fasting as a treatment by introducing it after a focal model of cerebral ischemia in rats and investigates its acute effects on infarct volume, BBB breakdown, and inflammation at 24 hours.“

2. The Results section requires improvement. I'm uncertain why the figure legends are placed within the Results section. Additionally, the authors should provide detailed explanations of the results instead of presenting them as brief, one-line statements.

PLOS One Journal asks the author to provide the Figure in the text of the manuscript, immediately following the paragraph in which the figure is first cited. Considering the comment on providing more detailed descriptions of the results, we extended the explanation of the results accordingly.

3. There are several significant previous studies in this field that have not been cited. I strongly recommend that the authors discuss these studies in both the introduction and the discussion sections, rather than solely focusing on a few particular studies. This will strengthen the overall narrative and emphasize the relevance of the current study in relation to other existing research.

We appreciate the reviewer’s comment and have expanded the references with relevant literature on fasting and ischemic stroke both, in the introduction and the discussion section.

4. The immunohistochemistry images appear unclear. It is necessary for the authors to provide clear and distinct images that clearly indicate the different cell types quantified in Figure 3. Additionally, it is advisable to include these images from all animals in the supplementary section for comprehensive visualization

We thank the reviewer for their comments, as both reviewers have highlighted the quality of the images provided we have updated these and provided higher-powered images for the revised manuscript.

1.	Abeysinghe HCS, Roulston CL. A Complete Guide to Using the Endothelin-1 Model of Stroke in Conscious Rats for Acute and Long-Term Recovery Studies. Methods Mol Biol. 2018;1717:115-33.

2.	Weston RM, Jones NM, Jarrott B, Callaway JK. Inflammatory cell infiltration after endothelin-1-induced cerebral ischemia: histochemical and myeloperoxidase correlation with temporal changes in brain injury. J Cereb Blood Flow Metab. 2007;27(1):100-14.

3.	Welniak-Kaminska M, Fiedorowicz M, Orzel J, Bogorodzki P, Modlinska K, Stryjek R, et al. Volumes of brain structures in captive wild-type and laboratory rats: 7T magnetic resonance in vivo automatic atlas-based study. PLoS One. 2019;14(4):e0215348.

4.	Swanson RA, Morton MT, Tsao-Wu G, Savalos RA, Davidson C, Sharp FR. A semiautomated method for measuring brain infarct volume. J Cereb Blood Flow Metab. 1990;10(2):290-3.
