# Supplementary material for: The effects of fasting on acute ischemic infarcts in the rat
Source: PLoS One. 2024 Dec 12;19(12):e0307313. doi: 10.1371/journal.pone.0307313 (PMC11637405; doi:10.1371/journal.pone.0307313)
Supplement: Attachment — Submitted filename: 20.06.2024 Response to Reviewers.docx [file pone.0307313.s004.docx]

**Response to Reviewers’ Comments**

**Reviewer #2:**

The authors have addressed my comments. While there is potential for additional experiments to delve deeper into the mechanistic details of the findings, this reviewer believes that this study can be beneficial to the scientific community, particularly for further exploration of the effects of fasting on stroke outcomes.

**Reviewer #3**:

The authors of this manuscript posit that fasting during the acute phase of cerebral infarction may lead to a reduction in the inflammatory response, thereby alleviating the impact of inflammation on cerebral infarction. To validate their hypothesis, the authors conducted rodent modeling trials. However, there are several aspects that require further consideration and elucidation by the authors.

*1. What is the success rate of endothelin-1-induced focal cerebral ischemia modeling in rats in this experimental procedure? What were the specific criteria employed to ascertain the success of the modeling process? Were any of the samples that failed to achieve successful modeling included in the treatment group?*

We thank the reviewers for their comments. The endothelin-1 stroke model is a well-validated and often-used method to induce ischemic stroke (1, 2). The stereotaxic microinjection of endothelin-1 induces a transient drop in blood flow lasting approximately 40 minutes and can be injected anywhere in the brain. This results in a focal lesion that can be used to study the effects of particular regions of the brain. We used cresyl violet staining for ischemia and immunohistochemical staining for blood-brain barrier integrity and neutrophil infiltration to directly and indirectly study focal lesions produced by endothelin-1. No animal had to be excluded in this study because of a potential failed lesion production. We amended the manuscript to make this clearer (lines 89-92).

*2. The rapamycin treatment group is mentioned in the Results section, yet no information regarding this group is provided in the Materials and Methods section of the manuscript.*

We are grateful to the reviewers for their valuable input. Although the original study involved comparing fasting, rapamycin treatment, and control groups, for this current publication, we have chosen to concentrate on our findings from the fasting group in comparison to the control group. The inclusion of rapamycin in the manuscript was an editing mistake, which has been rectified in the latest version.

**References**

1.	Biernaskie J, Corbett D, Peeling J, Wells J, Lei H. A serial MR study of cerebral blood flow changes and lesion development following endothelin-1-induced ischemia in rats. Magn Reson Med. 2001;46(4):827-30.

2.	Abeysinghe HCS, Roulston CL. A Complete Guide to Using the Endothelin-1 Model of Stroke in Conscious Rats for Acute and Long-Term Recovery Studies. Methods Mol Biol. 2018;1717:115-33.
